# Status of the Gastric Mucosa with Endoscopically Diagnosed Gastrointestinal Stromal Tumor

**DOI:** 10.1155/2014/429761

**Published:** 2014-07-02

**Authors:** Kouichi Nonaka, Shinichi Ban, Yoshimitsu Hiejima, Rei Narita, Michio Shimizu, Masayasu Aikawa, Ken Ohata, Nobuyuki Matsuhashi, Shin Arai, Hiroto Kita

**Affiliations:** ^1^Department of Gastroenterology, Saitama Medical University International Medical Center, 1397-1 Yamane, Hidaka, Saitama 350-1298, Japan; ^2^Department of Gastroenterology, NTT Medical Center Tokyo, 5-9-22 Higashi-gotanda Shinagawa-ku, Tokyo 141-8625, Japan; ^3^Department of Pathology, Saiseikai Kawaguchi General Hospital, 5-11-5 Nishikawaguchi, Kawaguchi, Saitama 332-8558, Japan; ^4^Graduate School of Healthcare, Tokyo Healthcare University, 3-11-3 Setagaya, Setagaya-ku, Tokyo 154-8568, Japan; ^5^Department of Pathology, Saitama Medical University International Medical Center, 1397-1 Yamane, Hidaka, Saitama 350-1298, Japan; ^6^Department of Surgery, Saitama Medical University International Medical Center, 1397-1 Yamane, Hidaka, Saitama 350-1298, Japan

## Abstract

*Background*. Since gastrointestinal stromal tumor (GIST) is a mesenchymal submucosal tumor, the endosonographic, CT, and MRI features of gastric GISTs have been widely investigated. However, the GIST-bearing gastric mucosa status has not been reported. *Objective*. To characterize the GIST-bearing gastric mucosa status in terms of the degree of inflammation and atrophy, assessed endoscopically. *Subjects and Methods*. The subjects were 46 patients with submucosal tumors (histologically proven gastric GISTs) who had undergone upper gastrointestinal endoscopy in our hospital between April 2007 and September 2012. They were retrospectively evaluated regarding clinicopathological features, the endoscopically determined status of the entire gastric mucosa (presence or absence and degree of atrophy), presence or absence and severity of endoscopic gastritis/atrophy (A-B classification) at the GIST site, and presence or absence of *H. pylori* infection. *Results*. Twenty-three patients had no mucosal atrophy, but 17 and 6 had closed- and open-type atrophy, respectively. Twenty-six, 5, 12, 1, 1, and 1 patients had grades B0, B1, B2, B3, A0, and A1 gastritis/atrophy at the lesion site, respectively, with no grade A2 gastritis/atrophy. *Conclusion*. The results suggest that gastric GISTs tend to arise in the stomach wall with *H. pylori*-negative, nonatrophic mucosa or *H. pylori*-positive, mildly atrophic mucosa.

## 1. Introduction

Gastric gastrointestinal stromal tumor (GIST) is a mesenchymal tumor arising from the muscularis propria of the gastric wall and is covered with normal mucosa, giving the endoscopic appearance of a submucosal tumor with bridging folds. Since gastric GIST is a submucosal tumor, its endosonographic, CT, and MRI features have been widely investigated [1–4]. However, the status of the GIST-bearing gastric mucosa itself has not been reported. Based on our experience, many GIST patients often show no evidence of *H*.* pylori* infection, gastritis, or mucosal atrophy on upper gastrointestinal endoscopy, and even* H*.* pylori*-infected patients may show mild gastritis in the background gastric mucosa. We consider that it is necessary to evaluate the GIST-bearing gastric mucosa using available data before testing the validity of this empirical rule and investigate the reason for it, if it is valid.

In this study, we retrospectively evaluated patients with submucosal tumors (histologically proven gastric GISTs) regarding the age, gender, site and size of the lesion, risk classification of GISTs, presence or absence and degree of endoscopic atrophy in the entire gastric mucosa, presence or absence and severity of endoscopic gastritis and mucosal atrophy at the lesion site, and presence or absence of* H. pylori* infection.

## 2. Subjects and Methods

**Figure 1:**
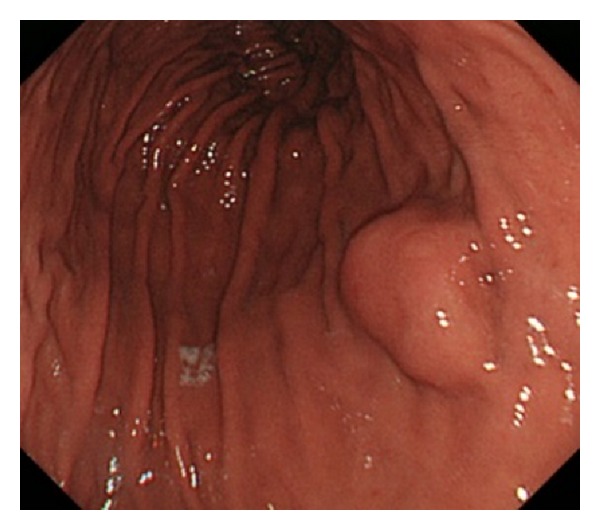
Endoscopic appearance of gastric mucosa of* H. pylori*- and atrophy-negative patient. Conventional endoscopic appearance. A 20 mm submucosal tumor was observed in the posterior wall of the middle gastric body. Conventional endoscopic findings were considered negative for* H. Pylori*.

**Figure 2:**
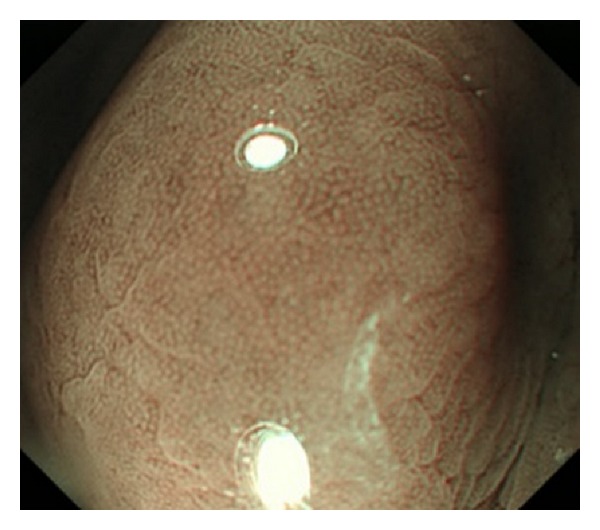
NBI-magnifying endoscopic findings. The lesion was classified as B0 according to Yagi's A-B classification system.

The subjects were 46 patients with submucosal tumors (histologically proven gastric GISTs) who had undergone both conventional (Figure 1) and NBI endoscopy (Figure 2) of the upper gastrointestinal tract in our hospital between April 2007 and September 2012. All subjects were derived from our hospital pathology database.

**Figure 3:**
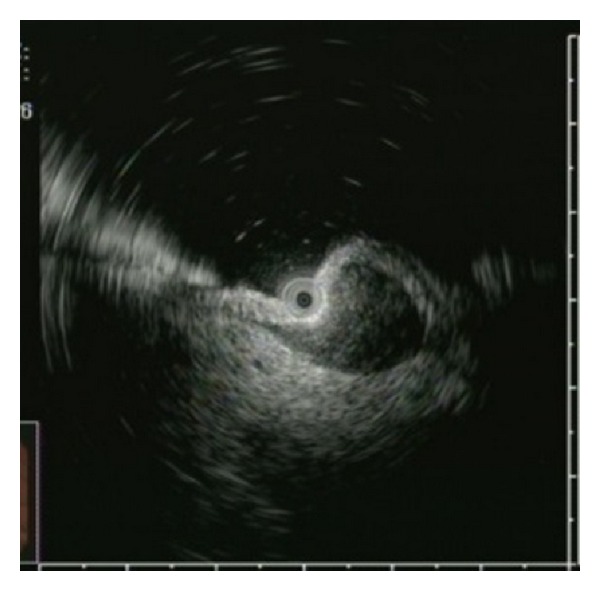
EUS findings. A homogeneous, hypoechoic tumor continuous with the fourth layer was observed.

All of them had undergone endoscopic ultrasonography: EUS (Figure 3). Of these patients, 40 had undergone surgical resection of the tumor, but 3 had not.

These 46 patients were retrospectively evaluated regarding the age, gender, site and size of the lesion, risk classification of GISTs, presence or absence and degree of endoscopic atrophy in the entire gastric mucosa, presence or absence and severity of endoscopic gastritis and atrophy at the lesion site, and presence or absence of* H. pylori* infection. The patients whose tumors were resected were evaluated regarding the maximum tumor diameter of the resected specimen, and those who did not undergo tumorectomy were assessed regarding the maximum tumor diameter determined by EUS. The patients were evaluated regarding the lesion site and risk classification of GISTs according to the 14th edition of the Japanese Classification of Gastric Carcinoma [5].

Endoscopic findings were evaluated by two experienced endoscopists (Kouichi Nonaka and Shinichi Ban). The presence or absence and grade of atrophy of the entire stomach were determined by conventional endoscopy and classified into nonatrophy and closed- and open-type atrophy according to the Kimura-Takemoto classification [6]. The presence or absence and severity of gastritis/atrophy were evaluated using Yagi's A-B classification system [7] based on the microsurface structure and microvascular architecture of the mucosa (Table 1). Yagi et al. defined types B0–B3 as magnified endoscopic images of the nonatrophic gastric mucosa, types A1-A2 as those of the atrophic mucosa, and type A-0 as that of the gastric antrum in* H. pylori*-noninfected patients. For endoscopists with the skills to perform conventional upper gastrointestinal endoscopic examination and diagnosis, it is straightforward to diagnose GISTs using these two classifications.

For the diagnosis of* H. pylori* infection, all patients had undergone at least one of the following tests: histological examination of endoscopic biopsy or surgical specimens, titration of serum anti-Hp IgG antibodies, and the urea breath test. The presence of at least one positive test was considered to indicate* H. pylori* infection.

Descriptive statistics were calculated for each variable.

## 3. Results

The mean age of the 46 patients was 63.9 years (range, 45–92 years). The male-to-female ratio was 0.92 (22 males and 24 females). Lesions were found in the upper, middle, and lower portions of the stomach of 32, 12, and 2 patients, respectively. The mean lesion diameter was 40.4 mm (range, 6–110 mm). GISTs were classified as very low, low, intermediate, and high risk in 8, 25, 7, and 3 patients, respectively. None of the patients had coexisting gastric adenoma or carcinoma.

Twenty-three patients had no mucosal atrophy, but 17 and 6 had closed- and open-type atrophy of the entire gastric mucosa, respectively. Twenty-six, 5, 12, 1, 1, and 1 patients had grades B0, B1, B2, B3, A0, and A1 gastritis and atrophy at the lesion site, respectively, and no patient had grade A2 gastritis and atrophy (Table 2).

In this study, 23 patients (50%) had evidence of* H. pylori* infection, and the gastric mucosa of 17 and 6 of them showed closed- and open-type atrophy, respectively (Table 3). However, 4, 5, 12, 1, 0, 1, and 0 patients had grade B0, B1, B2, B3, A0, A1, and A0 gastritis/atrophy at the GIST site, respectively, and the grade A1 gastritis/atrophy in the single patient was located in the gastric antrum. Thus, none of the patients had a clear pattern of mucosal atrophy. On the other hand, the 23 patients (50%) with no evidence of* H*.* pylori* infection coincided with the 23 patients whose entire gastric mucosa showed no evidence of atrophy (atrophy [0]), and the mucosa at the GIST site showed little or no inflammation/atrophy, as indicated by the findings of: B0 (22 patients), B1 (0), B2 (0), B3 (0), A0 (1), A1 (0), and A2 (0) (Table 3).

With advancing age, the rate of* H. pylori* infection increased, and the grade of mucosal atrophy tended to become higher (Table 4). Fifty-three percent (23/43) of the patients 50 years of age or older showed evidence of* H. pylori* infection.

## 4. Discussion

This study showed that most gastric GISTs arose in the upper and middle portions of or stomach, as in previous reports [8].

Since this study involved a small number of patients, further studies involving more patients are needed. However, in this study, 3 high-risk and 8 very low-risk patients based on the GIST risk classification system had* H*.* pylori*-negative, nonatrophic gastric mucosa, indicating no correlation between the presence or absence of mucosal atrophy and grade of GIST risk.

Twenty-three (50%) of the GIST patients and 53% (23/43) of those 50 years of age or older were found to be positive for* H. pylori* infection. In Japan, Shiota et al. reported that the* H*.* pylori* infection rate in patients 50 years of age or older for the period of 2002–2006 was 60% [9]. In 1992, Asaka et al. reported that the* H. pylori* infection rate in patients 40 years of age or older was 70% [10]; therefore, the* H. pylori* infection rate in patients 50 years of age or older in 2002, as estimated from these two studies, was about 60–70%.

Assuming that the* H. pylori* infection rate was about 60–70%, that rate of the GIST patients in this study was lower. Since some patients in this study were unable to undergo more than one test (e.g., tissue biopsy examination), they may have been false-negative for* H. pylori* infection. Although further studies involving more patients are needed, all of the 23 patients who were deemed* H. pylori*-positive had closed- or open-type atrophic gastritis on endoscopy, and all of the 23 patients who were deemed* H. pylori*-negative had* H. pylori*-noninfected gastric mucosa with no atrophy on endoscopy. Therefore, we suggest that no patients in this study were false-positive or false-negative for* H. pylori* infection.

The following analysis is based on the assumption, from the results of the above two studies, that the* H. pylori* infection rate in patients 50 years of age or older in Japan is about 60–70% [9, 10]. In a hypothetical case-cohort study, in which 43 GIST patients (≥50 years) in this study are compared to a cohort of patients (≥50 years) in Japan, the exposure-odds ratio is 1.3–2.0, suggesting that the incidence of GIST in* H. pylori*-negative patients will be 1.3–2.0 times as high as that in their* H. pylori*-positive counterparts.

In the 23 patients who had no evidence of* H. pylori* infection, the entire gastric mucosa showed little or no atrophy on endoscopy, and the mucosa at the GIST site exhibited little or no inflammation/atrophy, as indicated by the findings based on Yagi's A-B classification system. In the 23 patients with evidence of* H. pylori* infection, the gastric mucosa showed closed- or open-type atrophy, but the gastric mucosa at the GIST site showed grade B0 gastritis in the fundic glands of 4 patients, grade B1–B3 gastritis in 18 patients, no grade A atrophy, and a grade A1 lesion in the antral pyloric glands of 1 patient. In the 4* H. pylori*-positive patients with GIST in the fundic glands, the gastric mucosa at the GIST site was normal or mildly inflamed. Thus, in the patients studied, the gastric mucosa at the GIST site showed little or no atrophy.

These results suggest that gastric GIST arises from the muscularis propria underlying the* H. pylori*-negative, nonatrophic or* H. pylori*-positive, mildly atrophic gastric mucosa. None of the 46 patients had coexisting gastric adenoma or carcinoma. Although the incidence of gastric carcinomas and adenomas arising from* H*.* pylori*-negative, nonatrophic gastric mucosa without intestinal metaplasia is low, GISTs and other tumors may be present. Therefore, endoscopists should be prepared to perform endoscopy to avoid overlooking elevated lesions suggestive of submucosal tumors. Since this study involved a small number of patients in a single center, further studies involving more patients are needed to confirm our results.

## Figures and Tables

**Table 1:** Extended classification (A-B classification) of gastritis.

Magnified endoscopic image of nonatrophic mucosa	
B-0: uniform round pits (crypt openings: Cos) surrounded by a regular honeycomb subepithelial capillary network (SECN) and a regular arrangement of collecting venules seen as starfish-like structures (basic appearance of an* H. pylori*-negative patient).	
B-1: round pits (COs) surrounded by a regular or mild irregular honeycomb SECN; no collecting venules.	
B-2: round pits (COs) and dividing sulci; no normal SECN or collecting venules.	
B-3: dilated pits (COs) with more dense sulci; no normal SECN or collecting venules.	

Magnified endoscopic image of atrophic mucosa or gastric antrum	

A-1: ridged surface structures surrounding dilated, coiled subepithelial capillaries (SECs).	
A-2: villous to granular surface structures including irregular, narrowed, coiled SECs.	

Magnified image of *H. pylori-*noninfected gastric antrum	

A-0: a regular tubular mucosal pattern, along which a capillary network is seen. In some cases, arcuate white zones are surrounded by round or oval white zones.	

**Table 2:** Patient characteristics (*n* = 46).

Age	Mean: 63.9 years (45–92)
Sex (M : F)	22 : 24
Lesion site (U : M : L)	32 : 12 : 2
Lesion size	Mean: 40.4 mm (6–110 mm)
GIST risk classification	
Very low risk	8
Low risk	25
Intermediate risk	7
High risk	3
Status of atrophy of entire mucosa	
Atrophy (—)	23
Closed-type atrophy	17
Open-type atrophy	6
A-B classification of gastritis/atrophy at lesion site	
B0 : B1 : B2 : B3 : A0 : A1 : A2	26 : 5 : 12 : 1 : 1 : 1 : 0
*H. pylori* infection	
Positive : negative	23 : 23

**Table 3:** A-B classification of atrophy of the entire mucosa and status of the mucosa at the GIST site in *H. pylori-*positive and -negative patients.

	B-0	B-1	B-2	B-3	A0	A1	A2
*H. pylori*-negative (*n* = 23)							
Atrophy (—)	22				1		
C-type							
O-type							
*H. pylori*-positive (*n* = 23)							
Atrophy (—)							
C-type	4	4	8			1	
O-type		1	4	1			

C-type: closed-type atrophy, O-type: open-type atrophy.

**Table 4:** Rate of *H. pylori* infection and type of gastric mucosal atrophy by age group.

Age group	40–49	50–59	60–69	70–79	80–89	≥90
*H. pylori-*positive (%)	0/3	5/12	11/21	7/9	—	0/1
	(0)	(41.7)	(52.4)	(77.8)	—	(0)
Atrophy (—)/C-type/O-type	3/0/0	7/3/2	10/9/2	2/5/2	—	1/0/0

C-type: closed-type atrophy, O-type: open-type atrophy.
